# **Safety and Diagnostic Utility of Brain Biopsy and Metagenomics in Decision-Making for Patients with Inborn Errors of Immunity (IEI) and Unexplained Neurological Manifestations**

**DOI:** 10.1007/s10875-025-01878-y

**Published:** 2025-04-16

**Authors:** Jesmeen Maimaris, Julia Payne, Adriel Roa-Bautista, Judith Breuer, Nathaniel Storey, Sofia Morfopoulou, Alasdair Bamford, Felice D’Arco, Kimberly Gilmour, Kristian Aquilina, Jane Hassell, Yael Hacohen, Adikarige H.D. Silva, Ashirwad Merve, Thomas S. Jacques, Kanchan Rao, Robert Chiesa, Persis Amrolia, Juliana Silva, Helen Braggins, Jinhua Xu-Bayford, David Goldblatt, Austen Worth, Claire Booth, Winnie Ip, Waseem Qasim, Maaike Kusters, Marios Kaliakatsos, Julianne R Brown, Reem Elfeky

**Affiliations:** 1 Department of Immunology, Great Ormond Street Hospital for Children, NHS Foundation Trust, London, WC1N 3JH UK; 2 University College London (UCL) Institute of Immunity and Transplantation, London, UK; 3 Staffordshire Children’s Hospital at Royal Stoke, University Hospital of North Midlands NHS Trust, Royal Stoke University Hospital, Newcastle Road, Stoke-on-Trent, UK; 4 Microbiology, Virology and Infection Control, Great Ormond Street Hospital for Children, NHS Foundation Trust, London, WC1N 3JH UK; 5 Department of Infection, Immunity and Inflammation, University College London (UCL) Great Ormond Street Institute of Child Health, London, UK; 6 Deparment of Paediatric Infectious Diseases, Great Ormond Street Hospital for Children NHS Foundation Trust, London, UK; 7 Department of Neuroradiology, Great Ormond Street Hospital for Children, NHS Foundation Trust, London, WC1N 3JH UK; 8 Department of Paediatric Neurosurgery, Great Ormond Street Hospital for Children NHS Foundation Trust, London, WC1N 3JH UK; 9 Department of Neurosciences, Great Ormond Street Hospital for Children, NHS Foundation Trust, London, WC1N 3JH UK; 10 Department of Histopathology, Great Ormond Street Hospital for Children, NHS Foundation Trust, London, WC1N 3JH UK; 11 University College London (UCL) Great Ormond Street Institute of Child Health, London, UK; 12 Department of Blood and Bone Marrow Transplantation, Great Ormond Street Hospital for Children, NHS Foundation Trust, London, WC1N 3JH UK

**Keywords:** Primary immunodeficiency, Brain biopsy, Metagenomics, Inborn errors of immunity, Infection

## Abstract

Unexplained neurological symptoms can pose a diagnostic challenge in patients with inborn errors of immunity (IEI) where the aetiology can be varied, and diverse pathologies may require contrasting treatments. Brain biopsy, the process of sampling brain tissue directly, has historically provided histological and microbiological information and can now be exploited for deep metagenomic next generation analysis (mNGS). We conducted a retrospective analysis of clinical and diagnostic data on paediatric patients with IEI who had a brain biopsy between 2010 and 2022 at a UK tertiary centre where 14 patients fulfilled our search criteria. We report on clinical characteristics, adverse events and the additional impact of mNGS of brain biopsies, where these were conducted. We found that brain biopsy enabled diagnostics with manageable complications in most cases, either by tissue or metagenomics analysis (*n* = 11/14, 79%). We found that mNGS analysis improved the diagnostic yield of brain biopsy in 29% of IEI cases (*n* = 4/14). Brain biopsy enabled a change in management in 71% of cases (*n* = 10/14). This series provides compelling evidence for the safe and purposeful use of brain biopsy in children with IEI.

**Supplementary Information:**

The online version contains supplementary material available at 10.1007/s10875-025-01878-y.

## Introduction

Inborn errors of immunity (IEI) are associated with a range of neurological manifestations and complications [1–3]. Symptoms range from mild cognitive impairment and behavioural changes to severe, irreversible neurodisability and death [4, 5]. Neurological deterioration may present acutely or can be progressive, but either result in poor prognosis [6]. In patients with IEI, neurological sequelae may result from infection, inflammation and/or immune dysregulation [1, 4, 7]. Where the underlying pathology is unknown, treatment decisions are challenging, and diagnostic uncertainty contributes to delayed and/or suboptimal management, further compounding poor outcomes.

Key investigations used in unexplained neurological conditions include cerebrospinal fluid (CSF) analysis, cerebral Computed Tomography (CT) and Magnetic Resonance Imaging (MRI). Where these investigations are non-diagnostic, brain biopsy has been shown to be safe and can improve diagnostic yield [8, 9]. Previous paediatric cohort analyses from the UK and Canada have reported overall diagnostic yield of 69.4% and, up to 68.8%, respectively, and brain biopsy led to a change in management in 77.6% of cases [8, 9]. Metagenomic next generation sequencing (mNGS), the process by which deep sequencing of all RNA and DNA in a sample is used to detect evasive pathogens, can further enhance the diagnostic yield of biopsies and has a high clinical impact in immunocompromised patients [10–12].

There are limited data on the use of brain biopsy for children with IEI. The aim of our single-centre retrospective study was to evaluate the safety and efficacy of brain biopsy in this patient population.

## Methods

Full methods can be found in the supplemental section.

## Results

### Patient Characteristics

**Fig. 1:**
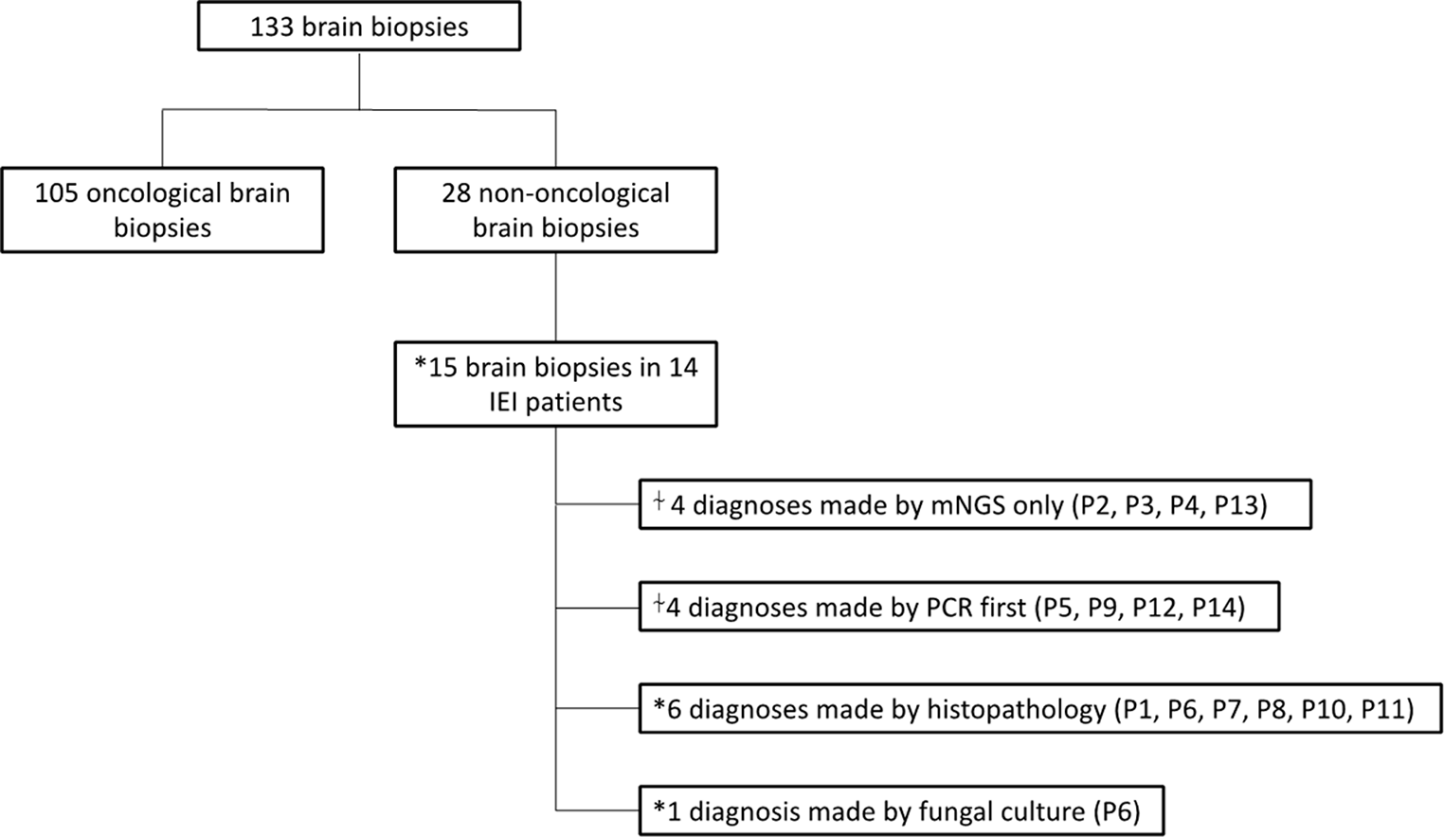
Overview of retrospective study analysis of patients who had brain biopsies between 2010–2022. * P6 had two biopsies: first diagnosis made by histopathology and second by fungal culture. ⍭ P12 and P14 detected by mNGS and PCR; PCR result was available first. mNGS – metagenomics next generation sequencing. PCR – polymerase chain reaction

133 patients had a brain biopsy at our centre between 2010 and 2022. 28 were non-oncological cases (*n* = 28/133), including 14 patients with IEI (*n* = 14/133) (Fig. 1). 5 patients had life-threatening or fatal neurosurgical complications as defined by Landriel Ibañez et al., including 1 patient with IEI (*n* = 5/133) (Tables 1 and 2) [13].

**Table 1:** Cases of patients with an inborn error of immunity (IEI) who had a brain biopsy/ies which led to change in diagnosis and management

Case	Age at biopsy	CNS clinical features	Differential diagnosis	Metagenomics utilised (Y/*N*)	Metagenomics pathogen detection (Y/*N*)	Pathogen detection site	IEI Diagnosis	CNS Diagnosis	Management changes following BB	Outcomes
P1	4 y	Focal afebrile seizures 3 intracerebral lesions	• Calcified metastases • CNS tuberculosis	N	N/A	N/A	P47phox CGD	Presumed fungal infection	Established diagnosis Anti-tuberculous therapy discontinued Anti-fungal (Caspofungin & AmBisome) commenced	Post HSCT Ongoing seizures and learning difficulty
P3 [1–3]	16 m	Irritability Dystonia Developmental regression	• Immune-mediated encephalitis • Viral encephalitis	Y	Y	Tissue Retrospectively detected by targeted PCR in CSF*	Cartilage hair hypoplasia	Astrovirus (HAstV-VA1/HMO-C-UK1)	Established diagnosis Palliative interventions commenced	Palliation Died 179 days post-biopsy
P5	4 y	Focal afebrile seizures 2 cerebral lesions with oedema	• EBV lymphoproliferation • Immune-mediated cerebritis • Malignancy	N	N/A	Tissue by targeted PCR Targeted PCR performed on CSF but not detected	CID (genetically undefined) with ectodermal dysplasia	EBV DLBCL	Established diagnosis Treatment with EBV-specific CTLs, IT Rituximab, IT & IV Methotrexate	Alive 7 years post- HSCT
P6	5 y	Confusion Focal seizures	• CNS HLH • Viral encephalitis	1st: Y	N	Tissue	XIAP deficiency	PRES	Showed no evidence of HLH Treated with steroids	Palliation Died 20 days 2nd biopsy
		Confusion Febrile seizures	• CNS *Aspergillus* infection	2nd: N	N/A	Tissue & CSF by fungal culture	XIAP deficiency	*Aspergillus fumigatus*	Established diagnosis Micafungin and voriconazole added to amphotericin	
P7	11 months	Afebrile seizures Multifocal enhancing lesions on MRI	• Fungal CNS infection • CNS lymphoma • CNS vasculitis	Y	N	N/A	SAP deficiency	CNS HLH	Established diagnosis Commenced on Alemtuzumab, etoposide and dexamethasone	Died 8 months post-HSCT
P8	4 y	Drowsiness Afebrile seizures	• CNS HLH • Viral encephalitis • CNS vasculitis	N	N/A	N/A	Genetically undefined EBV-HLH	CNS HLH	Established diagnosis HLH-94 protocol commenced	Died 22 days post-biopsy
P10	13 y	Occipital headache Paraesthesia Visual disturbance	• CNS vasculitis • Malignancy • CNS HLH	Y	N	N/A	Familial HLH due to perforin deficiency	CNS HLH	Established diagnosis HLH-94 protocol commenced	Alive 4 years post-HSCT
P11	3 y	Focal seizures Aphasia	• CNS HLH • Viral encephalitis • Inflammatory vasculitis	Y	N	N/A	SAP deficiency	CNS HLH	Established diagnosis Commenced on steroids and cyclophosphamide	Died 5 years post-HSCT
P12 [1]	13 m	Developmental regression Seizures	• Viral encephalitis • Inflammatory vasculitis	Y	Y	Tissue by targeted PCR & mNGS mNGS performed on CSF but pathogen not detected	MHC Class II deficiency due to *RFXAP* deficiency	Astrovirus VA1/HMO-C.	Established diagnosis Commenced ribavirin, nitazoxanide, favipiravir	Alive Continued dystonia and asymmetric tonic seizures
P13 [1]	16 y	Ptosis Seizures Progressive neuropathy Reduced GCS	• Viral encephalitis • CNS HLH • Inflammatory vasculitis	Y	Y	Tissue Targeted PCR retrospectively performed on CSF but not detected	Familial HLH due to Munc18-2 deficiency	Newcastle Disease Virus (*avian orthoavulavirus 1*)	Established diagnosis Immunosuppression withdrawn Commenced ribavirin, nitazoxanide, favipiravir	Died 6 months post-biopsy No significant neurological improvement noted
P14 [4]	13 y	Seizures Encephalopathy	• CNS HLH • Inflammatory vasculitis • Viral encephalitis	Y	Y	Tissue & CSF by targeted PCR & mNGS	Familial HLH due to Griscelli Syndrome type 2	HHV6 driven CNS HLH	Established diagnosis	Died 26 days post-biopsy

BB – brain biopsy, y- years, m- months, mNGS – metagenomic next generation sequencing, PCR – polymerase chain reaction, CGD – chronic granulomatous disease, HSCT – haematopoetic stem cell transplantation, SCID – severe combined immunodeficiency, EBV – Epstein-Barr virus, CID – combined immunodeficiency, DLBCL - Diffuse large B cell lymphoma, CTLs – Cytotoxic T lymphocytes, IT – Intrathecal, IV – Intravenous, CNS – Central nervous system, HLH - Haemophagocytic lymphohistiocytosis, XIAP - X-Linked Inhibitor of Apoptosis Protein, PRES - Posterior reversible encephalopathy syndrome, SAP - SLAM-associated protein, CSF – Cerebrospinal fluid, MHC – Major histocompatibility complex, HHV6 - Human herpesvirus 6

*Targeted PCR was not available at time of CSF sampling

**Table 2:** Cases of patients with an inborn error of immunity (IEI) who had a brain biopsy that did not lead to a change in management

Case	Age at biopsy	CNS clinical features	Differential diagnosis	Metagenomics utilised (Y/*N*)	Metagenomics pathogen detection (Y/*N*)	Pathogen detection site	IEI Diagnosis	CNS Diagnosis	Changes following BB	Outcomes
P2 [1, 5]	9 m	Dystonia Drop in GCS	• Viral encephalitis • Inflammatory vasculitis	Y	Y	Tissue *(No CSF available for retrospective targeted PCR)*	Common g-chain SCID	Coronavirus *OC43*	Extensive PCR did not find cause at the time. Coronavirus OC43 diagnosed retrospectively	Palliation Died 14 days post-biopsy
P4 [1, 6]	4 y	Seizures Visual loss Ataxia	• Viral encephalitis • Inflammatory vasculitis	Y	Y	Tissue (Targeted PCR restrospectively performed on CSF but not detected).	*RAG1* deficiency SCID	Attenuated Jeryl Lynn mumps virus (MuVJL5)	Extensive PCR did not find cause at the time. Mumps virus diagnosed retrospectively	Died 39 days post-biopsy
P9	15 y	Seizures Drop in GCS	• Fungal/ Parasitic CNS infection • Malignancy	N	N/A	N/A (Targeted PCR result from blood returned on same day as biopsy) (Targeted PCR performed on CSF and brain tissue, only detected in tissue.	LRBA deficiency	*Toxoplasma gondii*	Acute post-BB haematoma detected with midline shift New pontine & parietal haemorrhages	Died 1-day post-biopsy

BB – brain biopsy, m- months, y- years, PCR – polymerase chain reaction, GCS – Glasgow coma scale, SCID – severe combined immunodeficiency, RAG1 - recombination activating gene 1, CNS – Central nervous system, LRBA – Lipopolysaccharide-responsive beige-like anchor protein

15 brain biopsies were performed for these patients at our institution with 1 patient (P6) having two brain biopsies, a year apart. The first biopsy was for unexplained neurological symptoms and the second biopsy was to confirm central nervous system (CNS) *Aspergillus sp*. infection. The median time from the onset of neurological symptoms to the initial biopsy was 2.6 months (range: 10 days – 75 months). Brain biopsy led to a definitive diagnosis in 79% of cases (*n* = 11/14) and the biopsy results led to a change in management in 71% (*n* = 10/14).

Metagenomics was performed on 66% of biopsies (10/15). Three (3/10) biopsies had both RNA and DNA sequencing; seven (7/10) biopsies had RNA sequencing only as DNA sequencing was not available at the time of sampling for these patients. For one patient mNGS was performed after the patient’s death. Tissue samples were used for testing in all patients, and 2 patients underwent sequencing on both CSF and tissue samples. A causative organism was identified in 60% (6/10) by mNGS and in 40% (6/15) by both pathogen specific or broad range polymerase chain reaction (PCR). mNGS led to a change in management in 8 out of 10 biopsies where it was performed (80%).

Overall survival at 1-month post-biopsy was 67% (*n* = 10/15), reflecting the critical consequences of IEI, including one patient (P6) who died within 1 month of his second biopsy. One patient (P9) had a fatal biopsy-related complication that occurred 24 h post-biopsy. Patients were labelled chronologically according to the biopsy date from P1-P14. Details of each case are summarized below and in Tables 1 and 2.

### Adverse Events after Brain Biopsy

As standard care, patients who have undergone brain biopsy have a CT brain within 48 h to monitor for adverse events. Post-biopsy imaging was available for 79% (*n* = 11) of the patients. Radiological abnormalities were noted in 8 cases (*n* = 8/11; 73%). In 7 patients, these were minor changes in keeping with post-operative findings and, on clinical and radiological review, had no implications and no new symptoms were reported.

**Fig. 2:**
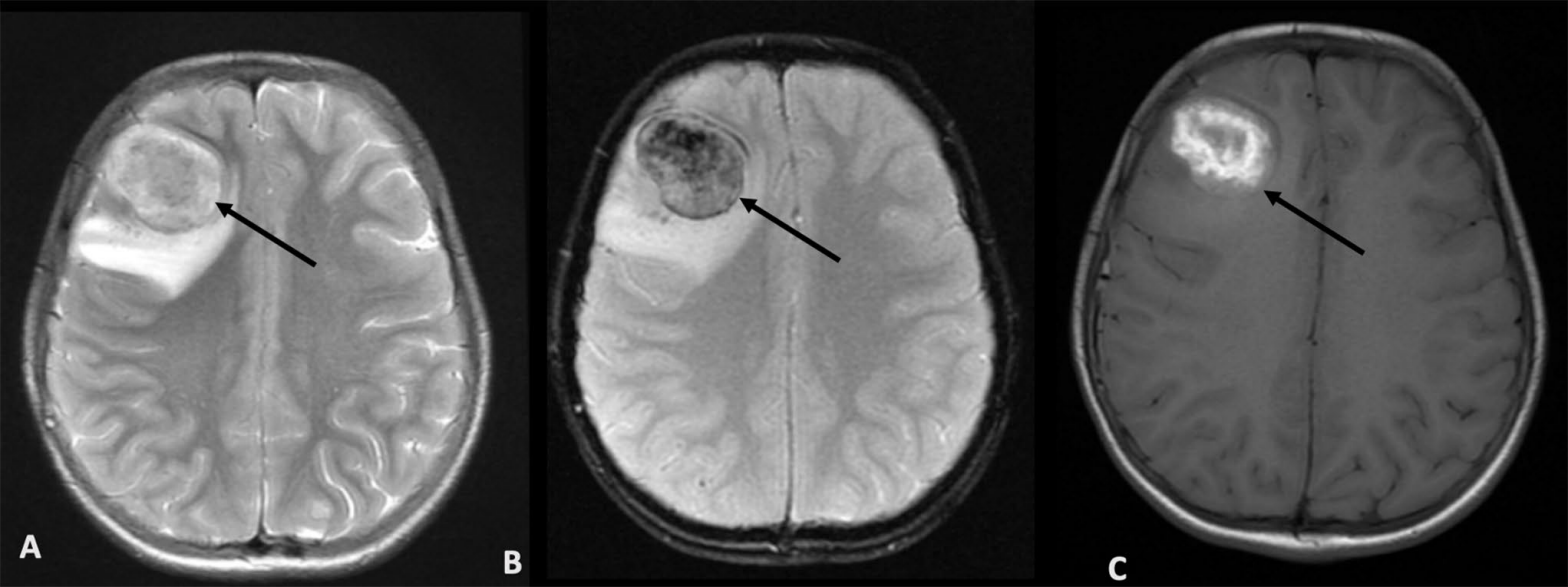
MRI brain images of P9 showing multiple ring-enhancing lesions with surrounding cerebral oedema. Axial T2 weighted images (**A**), axial T2 gradient (**B**) and axial pre-contrast T1 weighted images show a round parenchymal mass with internal T1 hyperintense signal (**C**) and T2 ‘blooming’ (**B**) which is in keeping with blood products. There is surrounding oedema and mass effect. MRI – magnetic resonance imaging

Patient 9 (P9) developed a large right intraparenchymal hematoma at site of the brain biopsy with midline shift. He was diagnosed at 14 years with Lipopolysaccharide-Responsive and Beige-like Anchor protein (LRBA) deficiency. Prior to onset of neurological symptoms, he had reduced intensity conditioning (RIC), matched unrelated donor (MUD), haemopoietic stem cell transplantation (HSCT), which was complicated by adenoviraemia, BK virus-related haemorrhagic cystitis, graft-vs-host disease (GVHD) and transplant-associated thrombotic microangiopathy (TA-TMA) requiring eculizumab. On day + 123, he developed fevers, seizures and reduced Glasgow coma score (GCS). MRI brain showed lesions suspicious for parasitic and/or fungal infection, prompting empiric initiation of sulfadiazine alongside broad-spectrum antibiotics and antifungal agents, based on imaging findings (Fig. 2). Due to persistently low GCS, he underwent a brain biopsy, while on the same day, blood PCR returned positive for *Toxoplasma gondii*. Prior to biopsy, platelet count of 117 × 10^9^/L. Following the biopsy, his platelet count dropped to 69 × 10^9^/L and then to 28 × 10^9^/L. He received a platelet transfusion, with a post-transfusion count of 129 × 10^9^/L. He had normal clotting studies throughout. Seven hours after the biopsy he was noted to have a dilated, sluggishly reactive right pupil and a haemoglobin drop from 107 to 97 g/L. CT brain revealed a right intraparenchymal haematoma at the site of the brain biopsy with midline shift. The haematoma was evacuated eight hours later, and post-surgery imaging showed that the midline shift had markedly improved with residual bilateral infratentorial swelling. However, new pontine and small left parietal haemorrhages were detected, not related to the biopsy site. Four hours after evacuation, his right pupil became fixed and dilated. CT brain showed that the acute pontine and left parietal haemorrhages were unchanged in size but his tonsils had descended by 7 mm with increasing swelling. Care was redirected to palliation after discussion with the family and the patient died shortly afterwards. Brain biopsy showed multifocal lesions in the cortex and white matter consisting of areas of vacuolation, necrosis, scattered basophilic debris (possible tachyzoites) and frequent cysts containing basophilic granules (resembling bradycysts of *Toxoplasma*). A few of the *Toxoplasma* parasites were associated with blood vessels which might explain the scattered areas of haemorrhage. Acute subarachnoid haemorrhage was also detectable from the biopsy. The brain tissue was positive for *Toxoplasma gondii* detected by PCR and thus, mNGS was not requested. Targeted PCR in CSF was negative for Toxoplasma.

### Utility of Brain Biopsy in our Study Cohort

For the remaining patients, we have divided the patients into 2 groups based on whether brain biopsy did or did not lead to diagnosis and change of management.

### 1) Patients where Brain Biopsy Led To a Change in Management

P1 was a 4-year-old girl who developed sudden-onset focal afebrile seizures with a background of cervical suppurative lymphadenopathy and culture-negative pericardial effusion (no diagnostic samples obtained). CNS imaging demonstrated focal partially calcified lesion with surrounding oedema, suggestive of malignancy. However, a subsequent brain biopsy showed granuloma formation (mNGS was not available). Dihydrorhodamine (DHR) flow cytometry test was performed, and she was diagnosed with chronic granulomatous disease (CGD). It was assumed that the brain lesion was fungal in aetiology, and she received empiric antifungal therapy before proceeding to RIC, matched sibling donor (MSD) HSCT. She is now 11 years post-HSCT with 100% donor chimerism but has ongoing seizures and learning disability.

P3, a 16-month-old male with cartilage hair hypoplasia, presented with recurrent infections and autoimmune neutropenia, requiring rituximab. He underwent a MUD HSCT complicated by adenoviraemia requiring a single dose of cidofovir and adenovirus-specific cytotoxic T lymphocytes (CTLs). On day 41 post-HSCT, he developed irritability and dystonia, progressing to loss of communication and motor skills and progressive hypotonia impacting airway control. MRI brain showed a global reduction in parenchymal bulk but excluded acute intracranial events. Broad range PCR in blood and CSF were negative for bacteria, fungi and viruses. However, brain biopsy revealed neuronal death with microglial activation, and tissue mNGS identified *Astrovirus (HAstV-VA1/HMO-C-UK1)* [8, 14]. This was subsequently detected in previously stored CSF by a specific *Astrovirus* PCR assay. The biopsy diagnosis enabled family discussion regarding prognostication and palliative care involvement, and P3 died 179 days post biopsy [8, 12, 14].

**Fig. 3:**
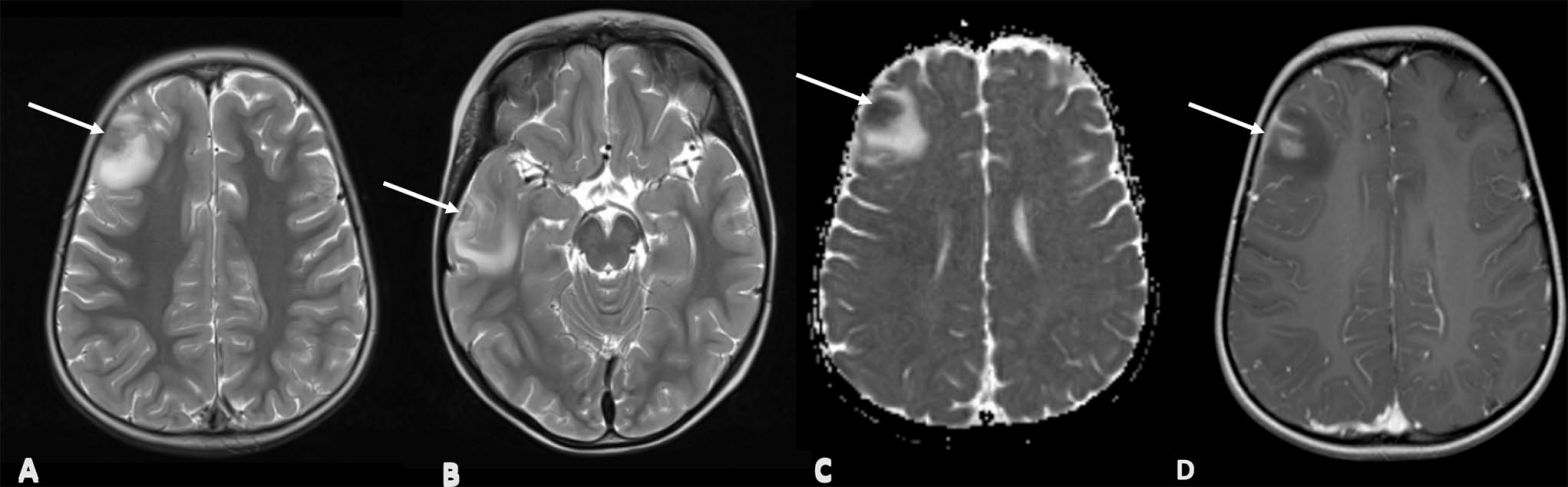
MRI brain images of P5 showing lesions in the right frontal and right temporal lobes with peripheral enhancement and surrounding oedema. Axial T2 weighted images (**A**, **B**), axial ADC diffusion map (**C**) and axial post-contrast T1 weighted images (**D**) show two lesions (arrows) in the right frontal and temporal lobes (arrows) with T2 hypointensity with diffusion restriction and enhancement in keeping with high cellularity expected in lymphoproliferative disorder. MRI – magnetic resonance imaging, ADC – apparent diffusion coefficient

P5 was a 4-year-old girl diagnosed with Epstein-Barr virus haemophagocytic lymphohistiocytosis (EBV-HLH) with a background of genetically undefined combined immunodeficiency and ectodermal dysplasia. She was treated with HLH-94 protocol [15] and rituximab but 2 months later, developed focal afebrile seizures with low-level serum EBV viraemia. MRI brain showed lesions in the right frontal and temporal lobes and CSF analysis was negative for EBV, but brain biopsy allowed confirmation of diffuse large B cell lymphoma (DLBCL) and tissue was EBV PCR positive (mNGS not obtained) (Fig. 3). She received DLBCL treatment followed by 8/10 mismatched unrelated donor (MMUD) cord HSCT. She remains well 7 years post-transplant with 100% donor engraftment.

**Fig. 4:**
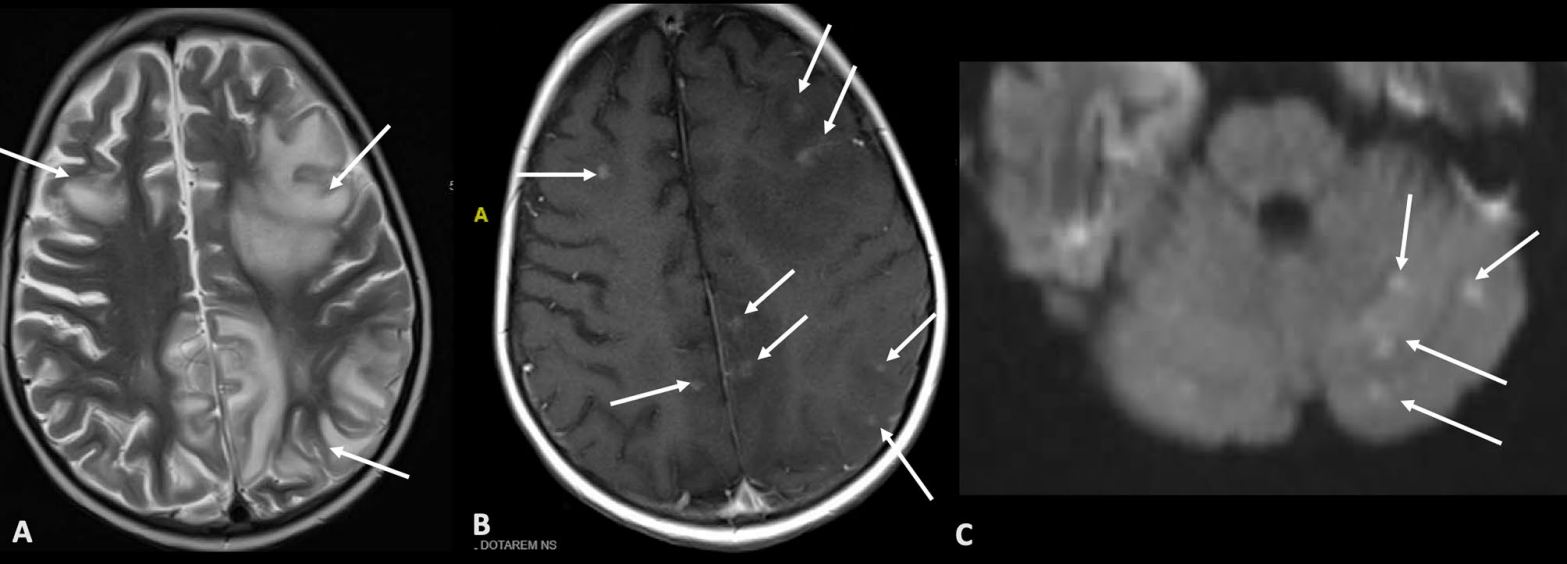
MRI brain images of P6 showing extensive bilateral disease throughout occipital, parietal and frontal lobe cortices. Axial T2 weighted images (**A**), axial post-contrast T1 weighted images (**B**) and axial diffusion weighted images (**C**) show multiple enhancing foci (arrows in **B**) with prominent surrounding oedema and show showing diffusion restriction (arrows in **C**). Imaging might suggest fungal infection. MRI – magnetic resonance imaging

P6 was diagnosed with X-Linked Inhibitor of Apoptosis Protein (XIAP) deficiency at 5-years old, after developing systemic EBV-HLH. He was treated with HLH-94 protocol [15] but 2 months into the treatment, he developed confusion, seizures and hypertension, thought to be secondary to posterior reversible encephalopathy syndrome (PRES). He developed features of systemic HLH and was restarted on dexamethasone and etoposide for suspected relapse of HLH. One month later, he deteriorated acutely with abnormal movements and non-convulsive status epilepticus. MRI brain showed extensive bilateral enhancing foci, but HLH and atypical infection could not be excluded (Fig. 4). A brain biopsy showed features of perivascular haemorrhage, astrocyte proliferation and perivascular macrophages but no conclusive evidence of HLH or infection was found, including mNGS. He proceeded to a fully matched HSCT but 10 months later, he developed seizures and had CNS imaging suggestive of infection. CSF results at the time were inconclusive. A repeat brain biopsy confirmed *Aspergillus fumigatus* on tissue culture (mNGS not required). Retrospective testing of the first tissue biopsy was negative for pan-fungal PCR and mNGS, although only RNA sequencing was performed at this time. Despite antifungal treatment, he developed progressive multisystem fungal disease and was referred to palliative care prior to death. He died 20 days following his second biopsy.

P7 was an 11-month-old boy with SLAM-Associated Protein (SAP) deficiency, who developed seizures without features of systemic HLH. MRI brain showed numerous areas of signal abnormality in subcortical white matter of both hemispheres, including in the internal capsule and cerebellum. Brain biopsy revealed an astrocytosis with focal oedema and perivascular infiltrate of T lymphocytes and macrophages. RNA sequencing of brain tissue excluded viral infection and he was diagnosed with CNS HLH. Alemtuzumab was given with partial remission followed by TCRαβ-deplete MMUD HSCT. Eight months later, he developed multisystem organ failure and died, likely secondary to uncontrolled transplant-associated thrombotic microangiopathy (TA-TMA).

P8 was a 4-year-old boy who was diagnosed with genetically undefined EBV-driven HLH. He was treated with the HLH-2004 protocol [16] but on day 7 developed generalized hyperreflexia with unprovoked clonus of his ankles. He was diagnosed with CNS HLH and started on HLH-94 protocol, with complete remission followed by 10/10 EBV-positive RIC MUD HSCT. Two months after HSCT, he had a sudden neurological deterioration with drowsiness and seizures. MRI brain/spine showed signal abnormality within the midbrain and swelling in the tegmentum with deep grey and white matter involvement, leptomeningeal enhancement, and areas of haemorrhage. He continued to clinically deteriorate, and brain biopsy was performed, showing CNS HLH with abnormal white matter, evidence of haemophagocytosis and few EBV-positive perivascular lymphocytes. Standard of care (broad range PCRs) performed on tissue were negative and further mNGS was not sought. CSF analysis found mixed chimerism with 10% donor and 90% recipient cells, while in bone marrow, there was 88% donor chimerism with no significant haemophagocytosis, confirming isolated CNS HLH relapse. HLH treatment was restarted but he had a poor response with evolution into systemic HLH. Unfortunately, he died 22 days after brain biopsy.

P10 was a 13-year-old boy who presented with suspected demyelinating disease characterized by recurrent headaches, visual changes, weakness and paraesthesia. He received intravenous immunoglobulin (IVIG) and methylprednisolone but continued to deteriorate neurologically. MRI brain showed multiple punctate foci of parenchymal enhancement in frontoparietal lobes, bilateral temporal-occipital lobes, corpus striatum, left thalamus, brainstem and both cellular hemispheres. A brain biopsy was performed, showing multiple foci of chronic inflammation, predominantly of T cells, with moderate numbers of macrophages showing microglial activation in parenchyma; all indicative of CNS HLH. This prompted targeted gene panel testing which found a diagnosis of perforin deficiency, the most common cause of familial HLH. He was treated with HLH-94 protocol followed by a MUD HSCT. He is alive and well 4 years post-transplant with 70–80% T cell donor engraftment.

**Fig. 5:**
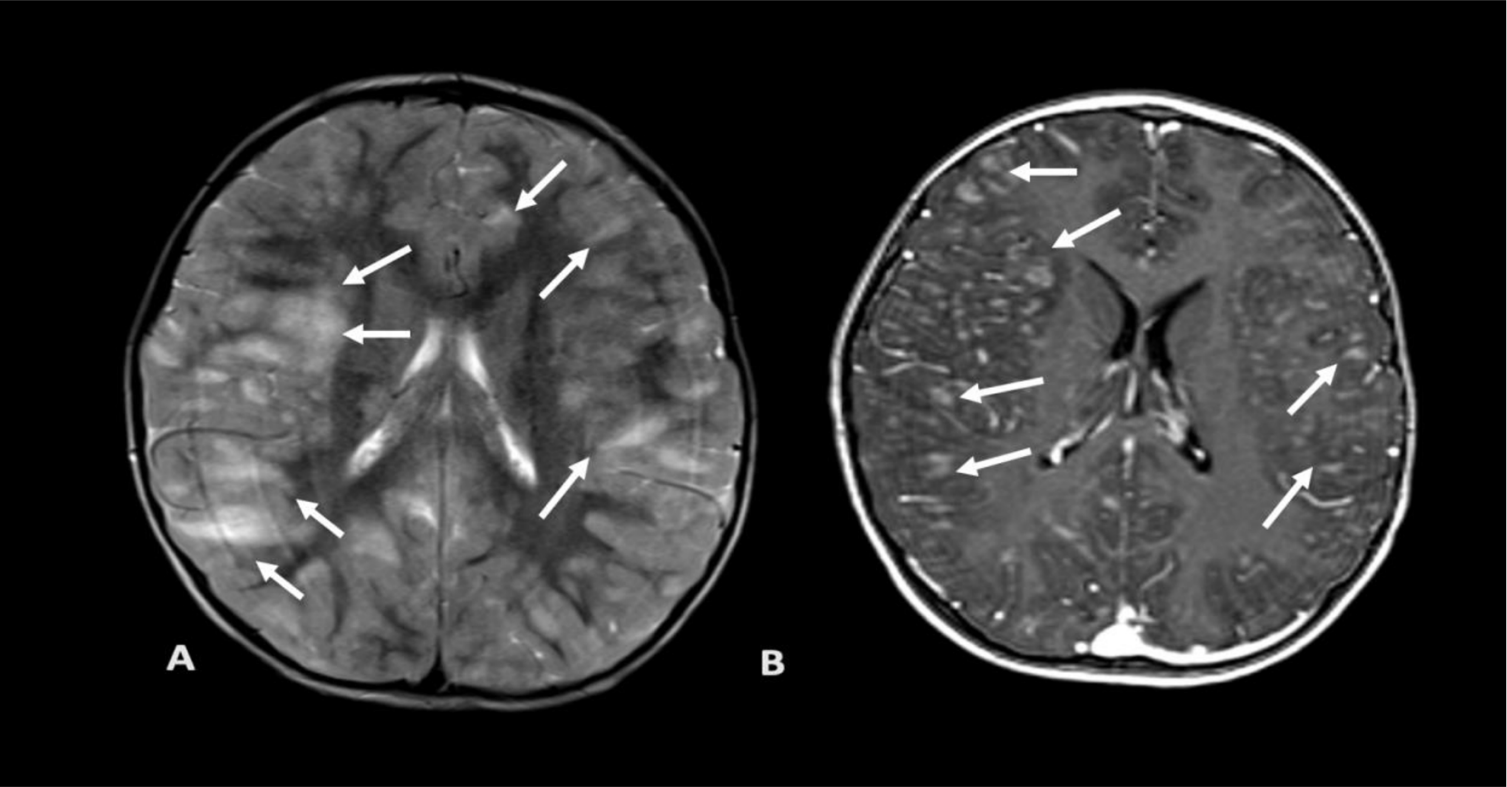
MRI brain images of P11 showing extensive supratentorial cortical and subcortical parenchymal damage with gliosis and white matter volume loss. Axial T2 weighted images (**A**), axial post-contrast T1 weighted-images (**B**) show uncountable enhancing lesions in the grey and white matter of all the cerebral hemispheres as well as infratentorial compartments (not shown). MRI – magnetic resonance imaging

**Fig. 6:**
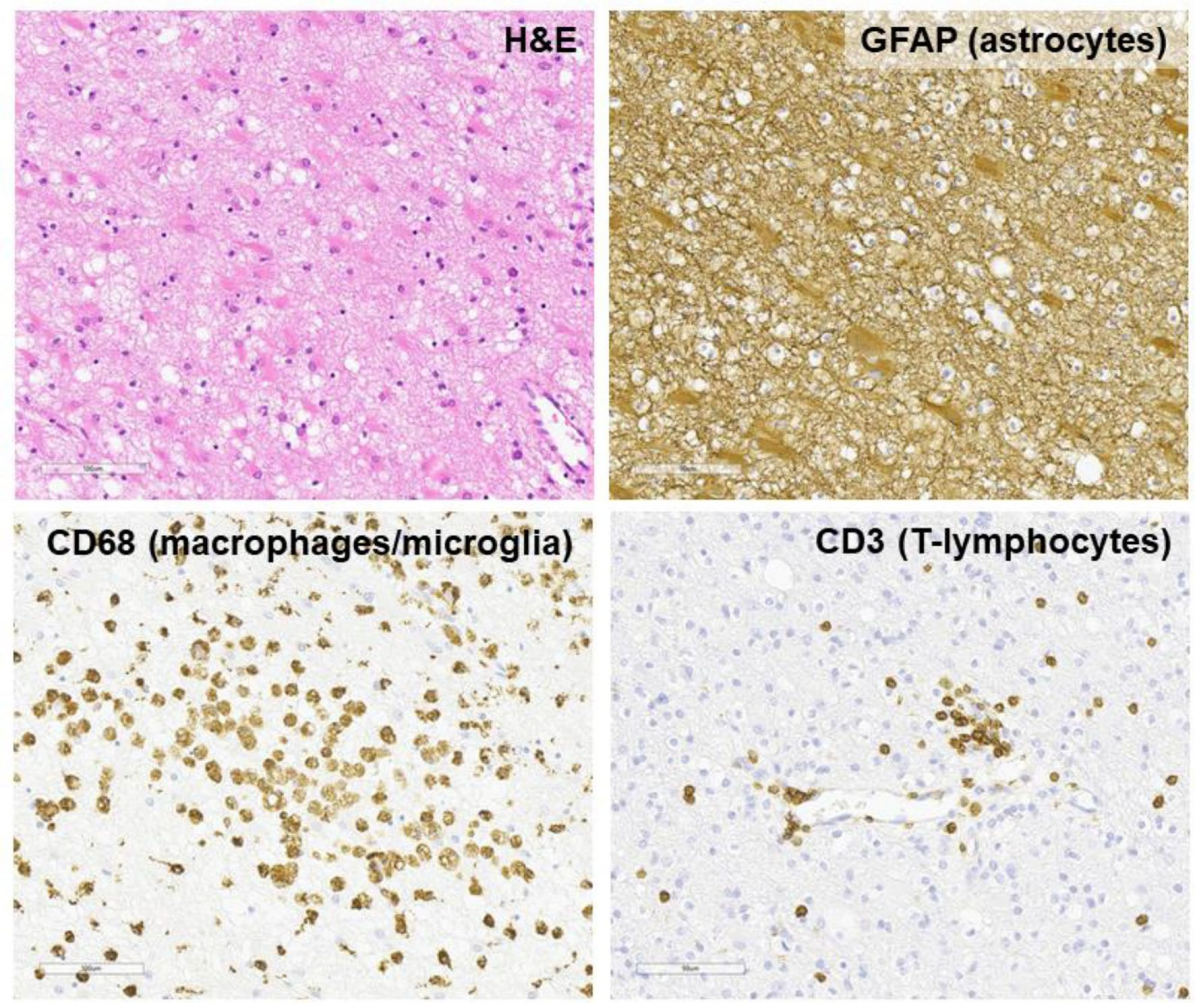
Histopathology of the second brain biopsy of P11, shows parenchymal damage with increased cellularity and gliosis, as highlighted by GFAP. There is microglial activation and macrophage infiltration as shown on CD68 and T-lymphocytes, particularly in the perivascular regions, on CD3. (H&E: haematoxylin and eosin; GFAP: glial fibrillary acidic protein, marker for glia; CD68 - marker for macrophages/microglia CD3 - marker of T-lymphocytes. Magnification: all images approximately x20)

P11 was a 3-year-old boy who developed lethargy, aphasia, focal seizures and behavioural disturbance, diagnosed as acute disseminated encephalomyelitis (ADEM). He was treated with IVIG, corticosteroids and cyclophosphamide but continued to neurologically deteriorate. At this time, he was diagnosed at his regional centre with SAP deficiency. MRI brain showed extensive parenchymal damage (Fig. 5). He underwent 2 brain biopsies: one with a regional clinical service and another in our institution. The first brain biopsy showed perivascular chronic inflammation with dense CD8 + T cell infiltrate affecting the cortex and white matter. His symptoms worsened and he was transferred to our hospital where a second brain biopsy was done, with mNGS performed. The tissue revealed extensive T-cell mediated perivascular inflammation, but infective pathogens were not detected by mNGS (Fig. 6). Exclusion of infection allowed treatment with corticosteroids, cyclophosphamide and IVIG, and he proceeded to HSCT with 100% whole blood donor chimerism and normal immune reconstitution. He continued to have residual neurodisability post-HSCT including aphasia. Unfortunately, he died 5 years later after developing aspiration pneumonia. Adenovirus was isolated by PCR from bronchial secretions and blood, though low levels.

**Fig. 7:**
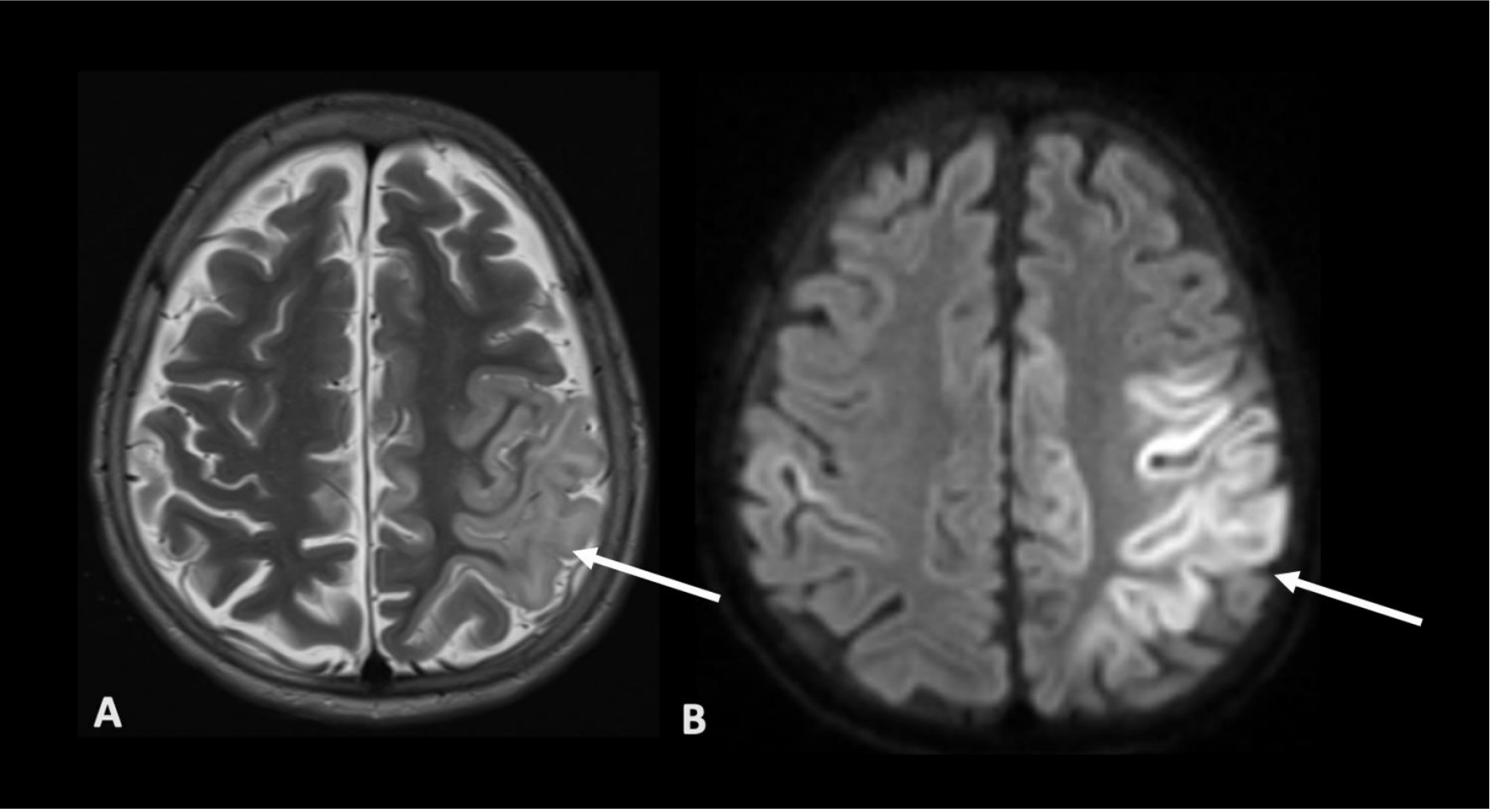
MRI brain images of P12 demonstrating features of generalised atrophy. Axial T2 weighted images (**A**) and axial diffusion weighted images (**B**) show extensive cortical signal change diffusion restriction and associated swelling in left posterior frontal and parietal regions. Similar changes were also present in the right hemisphere and lentiform nucleus. MRI – magnetic resonance imaging

P12 was a 13-month-old girl who presented with developmental regression and seizures. She had received her Measles/Mumps/Rubella (MMR vaccine) 1 month prior to neurological deterioration. Immunological work-up revealed pan-lymphopenia and she was diagnosed with MHC Class II deficiency. MRI brain showed generalized atrophy and brain biopsy showed cell loss and chronic inflammatory process with microglial activation and T cells, suspicious for viral encephalitis (Fig. 7). Tissue biopsy was tested simultaneously by targeted PCR and metagenomics; *Astrovirus VA1/HMO-C* was detected in both (PCR Ct 33). It is worth noting that targeted PCR for Astrovirus VA1/HMO-C is rarely available in clinical laboratories. *Astrovirus VA1/HMO-C* was not detected in CSF. She received triple antiviral therapy (ribavirin/nitazoxanide/favipiravir) which did not initially show radiological improvement however was associated with no further clinical deterioration. The clinical condition stabilised and slowly improved over the course of several months. After multiple family meetings, a decision was made not to proceed to HSCT. Four years post brain biopsy, P12 remains alive with slow neurological improvement [12].

**Fig. 8:**
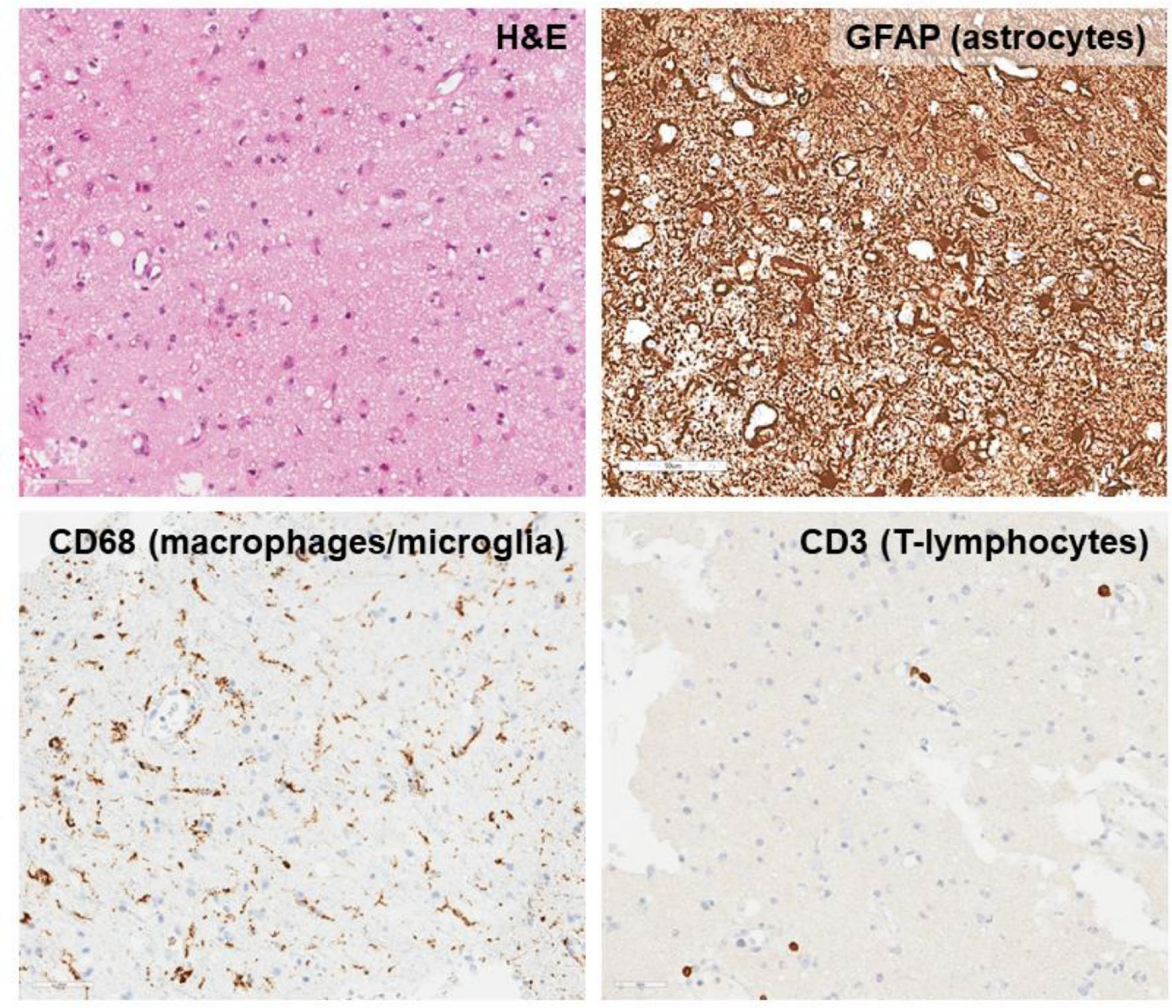
The superficial brain biopsy from P13 shows mild to moderate non-specific reactive changes including leptomeningeal thickening with reactive arachnoid proliferation and macrophage upregulation. There is gliosis, mild to moderate microglial activation and rare T-cells in the perivascular region. (Magnification – all images approximately x20)

P13 was a 16-year-old boy who 15 years prior underwent MMUD HSCT for familial HLH due to Munc18-2 deficiency and who developed sudden onset bilateral ptosis, seizures, progressive myopathy and neuropathy, requiring respiratory support due to a reduction in GCS. He had mixed donor chimerism (24% in whole blood, 16% T-lymphocytes and 28% granulocyte) and poor immune reconstitution with lymphopenia and hypogammaglobulinaemia. He received HLH-directed therapy but continued to deteriorate. MRI brain showed progressive widespread signal changes, partially sparing the right frontal, bilateral occipital, and right temporal lobes as well as significant bilateral cerebellar signal changes and involvement of both hippocampi. Brain biopsy demonstrated reactive changes (Fig. 8). mNGS of brain tissue detected Newcastle Disease Virus (*avian orthoavulavirus 1*). Upon retrospective testing, *avian orthoavulavirus 1* was not detected in CSF. Immunosuppression was withdrawn and antivirals commenced (favipiravir/nitazoxanide/ribavirin). P13 showed no clinical improvement and died 6 months after brain biopsy [12].

P14 was a 13-year-old male patient who presented acutely with sensorineural polyneuropathy. Six months later, he developed immune dysregulation and further seizures and encephalopathy in the setting of severe acute respiratory syndrome coronavirus 2 (SARS-COV-2) infection. Whole genome sequencing revealed the diagnosis of Griscelli Syndrome type 2 causing familial HLH. He received treatment for CNS-HLH including pulsed methylprednisolone (10 mg/kg) and ruxolitinib and after partial response, he developed acute neurological deterioration. MRI brain showed diffuse white matter signal change with associated swelling including pontine involvement and small cortical infarcts in the left temporal lobe. HLH systemic markers showed pancytopenia and ferritin of 1278ug/L with sparse haemophagocytosis on bone marrow and normal fibrinogen and liver function. Brain biopsy showed florid reactive astrogliosis and prominent histiocytic inflammation in a perivascular distribution. He had prominent CD68 and CD3 lymphocytic infiltrates with undefined granulomatous-type areas observed. Tissue biopsy was tested simultaneously by broad range PCR and mNGS; HHV-6 was detected in both (PCR cycle threshold (Ct) 20) with low-level HHV-6 viraemia detected in the blood concurrently. Based on a diagnosis of HHV-6-driven CNS-HLH, he started intense HHV-6-directed antiviral therapy and received no escalation in further immunosuppressive therapy. Unfortunately, he did not improve and progressed to systemic HLH prior to transition to palliative care and died 26 days after biopsy [17].

### 2) Patients where Brain Biopsy Did not Change Management

P2 was a 9-month-old boy diagnosed with common gamma chain severe combined immunodeficiency (SCID) following recurrent severe opportunistic lower respiratory infections. At 9 months, he developed irritability and poor feeding and was provisionally diagnosed with CNS viral encephalitis, however no pathogen was identified on blood or CSF cultures. CT brain showed non-specific white matter abnormalities. He received a 5/6 MMUD cord transplant without serotherapy or conditioning. On day + 26 post-transplant, he developed dystonic posturing and a drop in his GCS. MRI brain showed a global loss of white matter and delayed maturation of myelin. Brain biopsy showed abnormal cortex with vacuolation and frequent T lymphocytes in the leptomeninges and cortical parenchyma. Extended pathogen-specific PCR testing of the tissue was negative. He was referred to palliative care and died 14 days after his biopsy. mNGS was not available at the time of the brain biopsy but subsequent retrospective RNA sequencing identified Coronavirus HCoV-OC43 as the likely causative pathogen for his fatal encephalitis. CSF was not available for retrospective testing [12, 18].

P4 was 18 months old when he was diagnosed with recombination activating gene 1 (RAG1) deficiency SCID following recurrent severe respiratory tract infections. He had received the live MMR vaccine prior to diagnosis at 14 months. He had a CD3/CD19-deplete haploidentical HSCT with tyrosine kinase gene-modified donor lymphocyte infusion. In the following 24 months, he developed 2 episodes of suspected encephalitis: the first was a febrile illness with encephalitic changes on MRI, and the second 14 months later presented with seizure recurrence which both responded to antimicrobials, steroids and anticonvulsants. At 38 months post-HSCT, he developed further seizures with episodes of lethargy, disorientation, ataxia and visual loss. MRI brain showed progressive signal abnormality involving grey and white matter structures, specifically the basal ganglia and parieto-occipital region. Brain biopsy was suggestive of chronic encephalitis with neuronal loss, astrocytic gliosis, and chronic inflammatory cells, with no viral inclusions or granulomata. Despite treatment with broad-spectrum antimicrobials, IVIG and pulsed methylprednisolone, he deteriorated with developmental regression and further seizures. The patient received palliation and he died 39 days after his brain biopsy. Brain tissue mNGS completed after his death ultimately detected live-attenuated *Jeryl Lynn mumps virus (MuVJL5)* suggesting this as a likely cause for his symptoms. While retrospective testing of CSF by mumps targeted PCR was negative, targeted PCR in brain tissue confirmed the mNGS finding [12, 19].

## Discussion

This case series of 14 patients demonstrates the clinical utility of brain biopsy in the management of children with IEI with unexplained neurological symptoms. Brain biopsy led to a definitive diagnosis and change in management in 11 out of 14 cases and when performed early, facilitated timely diagnosis and initiation of treatment. In P1, early initiation of brain biopsy was essential for obtaining the underlying IEI diagnosis and allowed early and successful transplant prior to the accumulation of significant comorbidities. Where a diagnosis is not amenable to current therapies, brain biopsy findings can facilitate important management discussions around prognosis and palliation, as in P3, P12 and P14. In P11 and 14, earlier biopsy may have allowed earlier targeted management and prevented or limited irreversible and long-term neurological sequelae which ultimately contributed to mortality.

Timely mNGS of biopsy samples were instrumental in increasing the diagnostic yield of brain biopsy, particularly where CSF analysis with PCR was negative. For patients in whom only retrospective mNGS was possible (P2, P4), mNGS facilitated a diagnosis even if it could not directly impact management. In this cohort of patients, brain tissue provided a higher diagnostic yield than CSF, as found in Penner et al., [12]. Of the nine patients in whom a pathogen was identified in biopsy (6/9 by mNGS, 3/9 by PCR), six (6/9) had CSF tested either by metagenomics or targeted PCR for the respective pathogen. Five of these (5/6, 83%) did not have the pathogen detectable in CSF [20]. This is a small group of patients and there is inherent bias as generally patients in whom a diagnosis remains elusive are more likely to proceed to biopsy. Nonetheless we hypothesise our observation of higher tissue yield could be due to sampling of localised brain pathology or confinement of virus to the brain parenchyma therefore, testing CSF only could lead to a false-negative diagnosis [21]. Moreover, mNGS of CSF is more likely to detect incidental organisms not likely to be clinically significant [12]. CSF analysis and culture remain an important aspect of diagnosis and we advocate CSF analysis with PCR should be performed early where appropriate, particularly as PCR will often give a faster result than mNGS (1–2 days compared to 1–2 weeks).

mNGS allows for the detection of novel or unusual pathogens, an aspect particularly advantageous in patients with underlying IEI or immunosuppression [11, 14, 22–24]. Consequently, the diagnostic yield of mNGS is higher in immunocompromised patients in whom unusual, or rare or vaccine-related organisms are missed by standard diagnostic testing [12, 22, 23]. While it can be difficult to determine whether vaccine-related strains are bystanders or directly involved in pathology, underlying susceptibility to viruses is well described in patients with SCID [19, 25].

Whilst the utility of mNGS in immunocompromised patients is evident, clinical mNGS as part of standard of care remains restricted to relatively few laboratories with the necessary equipment and expertise. Most clinical mNGS services use lab-developed protocols that are costly, time-consuming and often require a bioinformatician for data analysis. There is a drive for standardisation and increased accessibility of mNGS protocols [26–29]. Increased implementation of laboratory automation and development of specialist staff expertise will allow wider adoption of mNGS across healthcare settings.

Three patients (P10, 11 and 14) presented with unexplained neurological features (ADEM-like) yet were ultimately diagnosed with CNS HLH. This highlights the importance of considering HLH in patients with a neurological presentation, even if they present in adolescence (P14). Early diagnosis of CNS-HLH is vital for timely treatment, and consequently, better outcomes. Where brain biopsy did not confirm the diagnosis, in some cases it allowed CNS infection to be deemed less likely and as such, more intensive immunosuppressive treatment to be given (as in the case of P7).

In this study brain biopsy had an excellent safety profile. One patient’s death (P9) occurred from a haematoma related to the biopsy site, however this patient had other simultaneous CNS bleeds which were not located near the biopsy site. He also had a history of TA-TMA. The brain biopsy found Toxoplasma associated with blood vessels and it is possible that the infection contributed to the bleeding. Coagulation profile and platelet numbers were normal pre-biopsy; however, this case highlights the need for caution and clear risk-benefit assessment of brain biopsy in patients with haematological comorbidities.

Our study collates a cohort of patients with IEI who had a brain biopsy following unexplained neurological symptoms. Limitations include the retrospective collection of data from a single paediatric centre. In rare cases, small biopsy samples may not represent the underlying pathology, a universal problem independent of the condition. In addition, safety and utility of brain biopsy are directly related to the experience and techniques employed by neurosurgeons and pathologists as well as rapid access to mNGS testing and this single-centre experience may not be generalisable for this reason.

## Electronic Supplementary Material

Below is the link to the electronic supplementary material.

Supplementary Material 1

## Data Availability

No datasets were generated or analysed during the current study.
