# Supplementary material for: Safety and Diagnostic Utility of Brain Biopsy and Metagenomics in Decision-Making for Patients with Inborn Errors of Immunity (IEI) and Unexplained Neurological Manifestations
Source: J Clin Immunol. 2025 Apr 16;45(1):86. doi: 10.1007/s10875-025-01878-y (PMC12003468; doi:10.1007/s10875-025-01878-y)
Supplement: Supplementary file 1 — Supplementary Material 1 [file 10875_2025_1878_MOESM1_ESM.docx]

**Supplemental Methods and Results**

**Methods**

Detailed laboratory results, imaging, and clinical documentation were reviewed for all patients under 18-years who were considered for brain biopsy at the Great Ormond Street Hospital (GOSH) in United Kingdom between 2010-2022. Only patients with an IEI diagnosis were included in the study. Data collected included clinical history, investigations, brain biopsy indication, result and outcomes. The study was performed in compliance with the Declaration of Helsinki.

**Targeted PCR**

Targeted PCRs were performed on brain biopsies as standard of care, depending on the clinical history of each patient. These are listed in Supplementary Table 1. For historical cases P1-P4 PCR was not widely applied to biopsies. All of these PCRs are accredited by the UK Accreditation Service (UKAS) to ISO15189:2022 standards and are provided by the diagnostic Microbiology and Virology laboratory at GOSH, unless otherwise indicated.

**Supplementary Table 1.** Targeted PCRs performed on patient brain biopsies as standard of care.

| **Case** | **Targeted PCRs performed** |
| --- | --- |
| P1 | Not performed |
| P2 | Not performed |
| P3 | Not performed |
| P4 | Not performed |
| P5 | Parechovirus, VZV, HSV-1, -2, Enterovirus, EBV, CMV, *Sreptococcus pneumoniae*, *Neisseria meningitidis*, 16S, ITS, *Mycobacterium tuberculosis* |
| P6 | Biopsy 1: *S. pneumoniae*, *N. meningitidis*, *S. aureus*, 16S, ITS |
|  | Biopsy 2: 16S |
| P7 | Parechovirus, Influenza A, VZV, Toxoplasma, HHV-6, HSV-1, -2, Enterovirus, EBV |
| P8 | Toxoplasma**, Leishmania**, Adenovirus, CMV, *Pneumoystistis jirovecii,* Parechovirus, Influenza A, B, Influenza H1N1, Parainfluenza 1, 2, 3, RSV A, B, Human metapneumovirus, Norovirus I, II, Rotavirus, Human Astrovirus, VZV, JC virus, BK virus, HSV-1, -2, Enterovirus, EBV, *M. tuberculosis, 16S, ITS* |
| P9 | Toxoplasma**, Adenovirus, CMV, Astrovirus VA1/HMO-C*, Parechovirus, VZV, JC virus, BK virus, HHV-6, HSV-1, -2, Enterovirus, EBV, *M. tuberculosis*, 16S, ITS, Influenza A, B, Influenza H1N1, Parainfluenza 1, 2, 3, RSV A, B, Human metapneumovirus |
| P10 | Parechovirus, Enterovirus, Adenovirus, CMV, VZV, HHV-6, HSV-1, -2, EBV, *S. pneumoniaie*, *S. aureus*, 16S, ITS |
| P11 | ITS, 16S |
| P12 | Astrovirus VA1/HMO-C*, Enterovirus, Parechovirus, Influenza A, B, Influenza H1N1, Parainfluenza 1, 2, 3, RSV A, B, Human metapneumovirus, Norovirus I, II, Rotavirus, Human Astrovirus |
| P13 | 16S, ITS, JC virus, BK virus, *M. tuberculosis* |
| P14 | Enterovirus, Astrovirus VA1/HMO-C*, Adenovirus, CMV, EBV, HHV-6, HSV-1, HSV-2, VZV, *S. aureus*, 16S, ITS, *M. tuberculosis*, NTM |

16S, 16S rRNA gene PCR (pan-bacterial); ITS, Internal Transcribed Spacer-2 PCR (pan-fungal); NTM, non-tuberculous mycobacteria

* Not UKAS accredited

** Send-away to an external diagnostic laboratory (not performed at GOSH)

**Brain biopsy technique**

Patients were biopsied using standard neurosurgical techniques. Large cortical superficial lesions, particularly if also involving the meninges, were biopsied using an open approach through a craniotomy. This allowed multiple samples to be obtained from a large lesion and a biopsy of the meninges at the same time. Deeper lesions were biopsied with a navigated system such as the Medtronic Stealth Vertek system (Medtronic PLC, Dublin, Ireland), which allows a precise image-guided biopsy with a tracked needle through a burr hole. Deep small lesions in eloquent locations e.g. brainstem, were biopsied with the Renishaw Neuromate robot (Renishaw PLC, Wootton-under-Edge, England, United Kingdom), which incorporated a stereotactic frame and afforded a high level of accuracy [1]. Prior to biopsy, patients undergo pre-operative optimisation including correction of abnormal coagulopathies, which may be related to bone marrow function and/or immunosuppressive treatment. Prophylactic procedure-related antimicrobial therapy may be considered, and is discussed and commenced prior to undertaking surgical intervention as required.

**Metagenomics**

Untargeted metagenomics (mNGS) for pathogen detection was performed by the clinical laboratory as part of routine care, as described previously [2]. Our clinical metagenomics service is accredited by the UK Accreditation Service (UKAS) to ISO15189:2022standards. RNA and DNA were extracted from fresh or frozen brain biopsy tissue within 1.5 hours. Separate libraries were prepared for DNA and RNA with depletion of host CpG-methylated DNA and host ribosomal RNA and sequenced on an Illumina NextSeq 550. Analysis was performed using metaMix to identify organisms present [3]. For quality assurance, every sample was spiked with MS2 RNA prior to nucleic acid purification, which must be detected in the sequence data for a valid result. Every batch of samples was accompanied by a positive and negative control, containing human DNA and RNA to mimic clinical specimens, Cowpox virus DNA and Feline Calicivirus (FCV) RNA. For a valid result, FCV and Cowpox was detected in the control sequence data, with no additional organisms detected. For a result to be valid, at least 10 reads were detected from the identified organism, mapping to three or more regions of the genome and confirmed by BLAST analysis of the mapped consensus sequence.

**Results**

There were no false positive detections by mNGS.

Metagenomics genome coverage statistics

| Patient (reference) | Pathogen | Genome coverage (%) | Average read depth |
| --- | --- | --- | --- |
| P2 [4] | Coronavirus OC43 | 100 | 3864 |
| P3 [5] | Astrovirus VA1 | 22 | 7 |
| P4 [6] | Jeryl Lynn mumps virus (MuVJL5) | 99.94 | 290 |
| P12 | Astrovirus VA1 | 100 | 61704 |
| P13 | Avian paramyxovirus | 100 | 3849 |
| P14 | Human herpesvirus 6 | 99 | 25 |

*100% subsequently generated by capillary sequencing of tiled PCR amplicons

Data availability

Metagenomics non-human microbial read data (excluding P6 for whom raw data is no longer available) is available on SRA: <http://www.ncbi.nlm.nih.gov/bioproject/1198490>.

Bioproject accession number: PRJNA1198490

| Sample ID | Patient | Source | Accession |
| --- | --- | --- | --- |
| P2_PLYUTRX7R6_RNA | P2 | Tissue, Brain | SRR31740090 |
| P3_NZIOLKREOL_RNA | P3 | Tissue, Brain | SRR31740089 |
| P4_J2RF4TAYZT_RNA | P4 | Tissue, Brain | SRR31740083 |
| P7_PDMQUP2YJ6_RNA | P7 | Tissue, Brain | SRR31740082 |
| P10_PQT092KFPL_RNA | P10 | Tissue, Brain | SRR31740081 |
| P11_M4QY6Y5RT4_RNA | P11 | Tissue, Brain | SRR31740080 |
| P12_72DMJ09D89_RNA | P12 | Tissue, Brain | SRR31740079 |
| P12_MDCTWS9MM1_DNA | P12 | CSF | SRR31740078 |
| P12_MDCTWS9MM1_RNA | P12 | CSF | SRR31740077 |
| P13_VS2T0RPNNF_DNA | P13 | Tissue, Brain | SRR31740076 |
| P13_VS2T0RPNNF_RNA | P13 | Tissue, Brain | SRR31740088 |
| P14_TRAG281R3L_DNA | P14 | Tissue, Brain | SRR31740087 |
| P14_TRAG281R3L_RNA | P14 | Tissue, Brain | SRR31740086 |
| P14_WSNB7I7D31_DNA | P14 | CSF | SRR31740085 |
| P14_WSNB7I7D31_RNA | P14 | CSF | SRR31740084 |

References:

1. Marcus, H.J., et al., *Robot-assisted stereotactic brain biopsy: systematic review and bibliometric analysis.* Childs Nerv Syst, 2018. **34**(7): p. 1299-1309.

2. Atkinson, L., et al., *Untargeted metagenomics protocol for the diagnosis of infection from CSF and tissue from sterile sites.* Heliyon, 2023. **9**(9): p. e19854.

3. Morfopoulou, S. and V. Plagnol, *Bayesian mixture analysis for metagenomic community profiling.* Bioinformatics, 2015. **31**(18): p. 2930-8.

4. Morfopoulou, S., et al., *Human Coronavirus OC43 Associated with Fatal Encephalitis.* N Engl J Med, 2016. **375**(5): p. 497-8.

5. Brown, J.R., et al., *Astrovirus VA1/HMO-C: an increasingly recognized neurotropic pathogen in immunocompromised patients.* Clin Infect Dis, 2015. **60**(6): p. 881-8.

6. Morfopoulou, S., et al., *Deep sequencing reveals persistence of cell-associated mumps vaccine virus in chronic encephalitis.* Acta Neuropathol, 2017. **133**(1): p. 139-147.
